# Effects of Abscisic Acid and Salicylic Acid on Gene Expression in the Antiviral RNA Silencing Pathway in Arabidopsis

**DOI:** 10.3390/ijms20102538

**Published:** 2019-05-23

**Authors:** Mazen Alazem, Kook-Hyung Kim, Na-Sheng Lin

**Affiliations:** 1 Department of Agricultural Biotechnology, College of Agriculture and Life Sciences, Seoul National University, Seoul 08826, Korea; m.alazem@gmail.com; 2 Plant Genomics and Breeding Institute, College of Agriculture and Life Sciences, Seoul National University, Seoul 08826, Korea; 3 Institute of Plant and Microbial Biology, Academia Sinica, Taipei 11529, Taiwan; 4 Research Institute of Agriculture and Life Sciences, College of Agriculture and Life Sciences, Seoul National University, Seoul 08826, Korea

**Keywords:** abscisic acid, salicylic acid, crosstalk, RNA silencing pathway, bamboo mosaic virus

## Abstract

The RNA silencing pathways modulate responses to certain stresses, and can be partially tuned by several hormones such as salicylic acid (SA) and abscisic acid (ABA). Although SA and ABA are often antagonistic and often modulate different stress responses, they have similar effects on virus resistance, which are partially achieved through the antiviral RNA silencing pathway. Whether they play similar roles in regulating the RNA silencing pathway is unclear. By employing coexpression and promoter analyses, we found that some ABA- and SA-related transcription factors (TFs) are coexpressed with several *AGO*, *DCL*, and *RDR* genes, and have multiple binding sites for the identified TFs in the queried promoters. ABA and SA are antagonistic with respect to the expression of *AGO1* and *RDRs* because ABA was able to induce these genes only in the SA mutant. Nevertheless, both hormones showed similarities in the regulation of other genes, for example, the induction of *AGO2* by ABA was SA-dependent, indicating that ABA acts upstream of SA in this regulation. We inferred that the similar effects of ABA and SA on some genes resulted in the redundancy of their roles in resistance to bamboo mosaic virus, but that the two hormones are antagonistic with respect to other genes unrelated to their biosynthesis pathways.

## 1. Introduction

Plants employ hormones to tune their responses to environmental stimuli or developmental changes. Several hormones, including abscisic acid (ABA) and salicylic acid (SA), exhibit antagonistic interrelations that help plants balance their responses in order to limit the associated fitness costs. ABA is well-known for its role in plant responses to abiotic stress and for its multifaceted roles in plant–pathogen interactions [1,2,3]. SA, in contrast, has long been known to regulate plant defenses against biotrophs by priming systemic acquired resistance [4]. 

Although ABA and SA exhibit antagonistic interrelations in response to biotic and abiotic stresses [5,6,7,8], a few reports indicated that they share some integrative effects in modulating specific responses. In guard cells, for example, the SA signaling pathway merges with the ABA signaling pathway through Ca^2+^-dependent protein kinases to regulate stomatal closure [9]. Regarding resistance to viruses, both hormones have been reported to increase plant resistance to several pathogenic viruses partially through the RNA silencing pathway [10,11,12,13]. For instance, ABA regulates the expression of argonautes (AGO) *1*, *2*, *3*, and *4* and the microRNA *168a* [3,14], and induces resistance to infection by bamboo mosaic virus (BaMV), mainly through its effects on *AGO2* and *AGO3* [3]. ABA and *AGO2* are also involved in resistance to potato virus X (PVX) in *Arabidopsis thaliana*. PVX replicates very weakly in *A. thaliana* due to the effect of AGO2 on resistance [15], and impairment of the ABA pathway reduces the accumulation of *AGO2* and allows PVX to accumulate and move systemically [3]. SA also induces the expression of RNA-dependent RNA polymerase (*RDR) 1* in *A. thaliana* and contributes in resistance to plum pox virus by enhancing the production of virus-derived short-interfering RNAs (vsiRNAs) [13,16,17]. Nevertheless, the effects of ABA and SA on the antiviral RNA silencing pathway are incompletely understood, and there is little information of whether or how these hormones affect the majority of the genes involved in this pathway. The key genes in the RNA silencing pathway include the dicer-like (*DCL*), *RDR*, and *AGO* gene families [18,19]. There are several other genes that maintain the integrity of the RNA silencing pathway [18,19]. However, the diversity and the proposed redundancy of several genes in the AGO, DCL, and RDR families have motivated us to investigate the involvement of these genes in ABA- and SA-mediated effects in Arabidopsis.

Given that ABA and SA are antagonistic to each other in response to abiotic stress and nonviral infections (bacterial and fungal), but share some similarities in inducing resistance/tolerance to viral infections; the objective of the current research was to determine how SA and ABA regulate genes involved in the antiviral RNA silencing pathway and whether the two hormones act in a parallel, antagonistic, or hierarchical manner in regulating the targeted genes. To accomplish this, we used PlantPan 2.0 to conduct coexpression analyses of SA- and ABA-related transcription factors (TFs) that may regulate genes in the *AGO*, *DCL*, and *RDR* families. PlantPan 2.0 contains a large number of experimentally verified TF matrices as well as coexpression profiles of TFs and their target genes under various conditions [20]. We measured the expression of *AGO*, *DCL*, and *RDR* genes in SA- or ABA-treated wild type (WT) *A. thaliana* seedlings and in seedlings of the SA-mutant *sid2-1*, ABA-mutant *aao3*, and their double mutant *sid2-1:aao3*. Finally, we determined how impairment of the SA and ABA pathways affects plant susceptibility to BaMV, which is a potexvirus [21] with a positive-sense single-stranded RNA genome [22,23] that is packed in flexible filamentous particles [24]. To study plant–virus interaction, most researchers have used mature plants (usually 25 days old for Arabidopsis) and have collected samples 5 to 20 days postinfection. As a consequence, it remains unclear how younger plants respond to virus infection and hormone treatments. In this study, we used 6-day-old *A. thaliana* seedlings to investigate the effects of ABA and SA on the antiviral RNA silencing pathway. Our results showed that ABA and SA have similar positive effects on the expression of several *AGO* genes, with one exception (ABA-induction of *AGO2* was found to be SA-dependent). On the other hand, both hormones exhibited mutual antagonism of *AGO1* and *RDR* expression in that ABA clearly induced those genes in the SA mutant *sid2-1*. The effects of these hormones on the expression of RNA silencing genes might be spatiotemporally regulated because the responses of some genes differ depending on tissue and plant age.

## 2. Results

### 2.1. Binding Sites of Several TFs Regulated by ABA and SA Are Located in the Promoter Regions of Genes Belonging to the AGO, DCL, and RDR Families

PlantPan 2.0 provides a large number experimentally verified matrices of TFs and their binding sites. This tool employs microarray expression data obtained from plants under different biotic/abiotic stresses, at different developmental stages, and under various hormone treatment conditions (Table S2) [20]. These public microarray data are compiled in the ExPath 2.0 expression database [25], and, because it offers coexpression analyses, ExPath can be used to determine whether genes are coexpressed under any specific condition.

When hormone treatment or biotic-stress conditions were selected, our coexpression analyses revealed that several ABA- or SA-related TFs can be coexpressed with genes in the AGO, DCL, and RDR families (Table 1). Almost all of these genes were determined to have at least two coexpressed TFs that are ABA- or SA-regulated. Only *RDR6* lacked an SA-related coexpressed TF. Except for SA-regulated TGA2, TGA4, TGA6, and OBP1, there was no unified set of TFs that could bind to the same genes (Table 1). Most of the identified SA- and ABA-related TFs have positive regulatory roles in their corresponding signaling pathway; the exceptions are ATAF1 and HB33, which have negative roles in the ABA signaling pathway [26,27,28] (Table 1). The identified TFs modulate ABA- or SA-mediated responses depending on the tissue, developmental stage, or stress condition. These data suggest that, depending on the type of stress, ABA or SA may regulate plant response partially/collaboratively through the RNA silencing pathway. It should be noted that PlantPAN 2.0 lacks coexpression profiles for *AGO6* due to the absence of a probe targeting *AGO6* in the Affymetrix GeneChips microarray sourced from the Arabidopsis information resource (TAIR) database. In addition, we could not detect any expression of *AGO5* and *AGO9* in the 8-day-old mock or hormone-treated seedlings using several pairs of primers (data not shown). This was confirmed using the Arabidosis eFP browser, which offers expression data of Arabidopsis genes at any developmental stage (http://bar.utoronto.ca/efp2/). 

**Figure 1:**
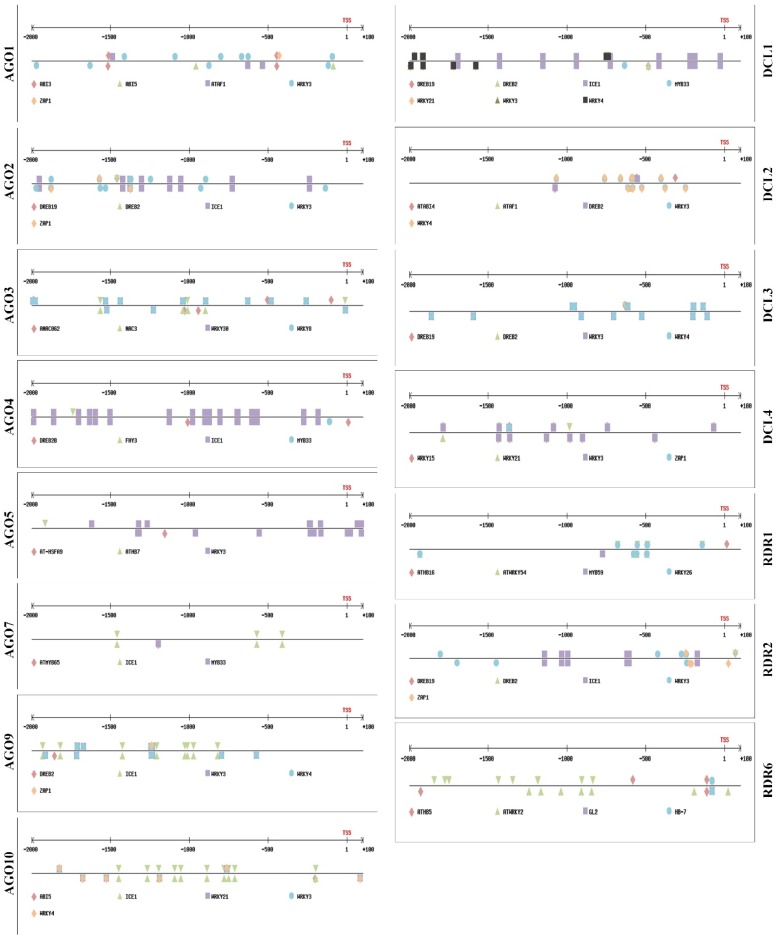
Transcription factor (TF) binding sites of ABA- and SA-related TFs in the promoters of RNA silencing genes: Binding sites of ABA- and SA-regulated TFs on the promoters of RNA silencing genes (*AGO*, *DCL*, and *RDR* gene families). Promoter regions 2000 bp upstream of the transcription start site (TSS) were subjected to promoter analyses using the Plant Promoter Analysis Navigator in PlantPan 2.0. The TFs and their binding sites (*cis*-regulatory elements) were visualized using PlantPan 2.0.

Next we scanned the promoter regions of *AGO*, *DCL*, and *RDR* genes for transcription factor binding sites (TFBSs) of the previously identified TFs. The scan showed that all promoters have multiple binding sites for the identified TFs, indicating that these genes can be regulated by both ABA and SA (Figure 1). Because the TFs TGA2, TGA4, TGA5 TGA6, ICE1, and HB33 have several binding sites in the promoters of several genes, the TFBSs were separately visualized (Figure S1).

### 2.2. ABA and SA Exert Similar Effects on Expression of Several Genes in the RNA Silencing Pathway 

To examine the effects of ABA and SA on the expression of *AGO*, *DCL*, and *RDR* genes, we treated the seedlings of the WT, the SA mutant *sid2-1*, the ABA mutant *aao3*, and the ABA-SA double mutant *sid2-1:aao3* with ABA, SA, or mock. Seedlings were collected two days after hormone treatment for analysis of gene expression.

**Figure 2:**
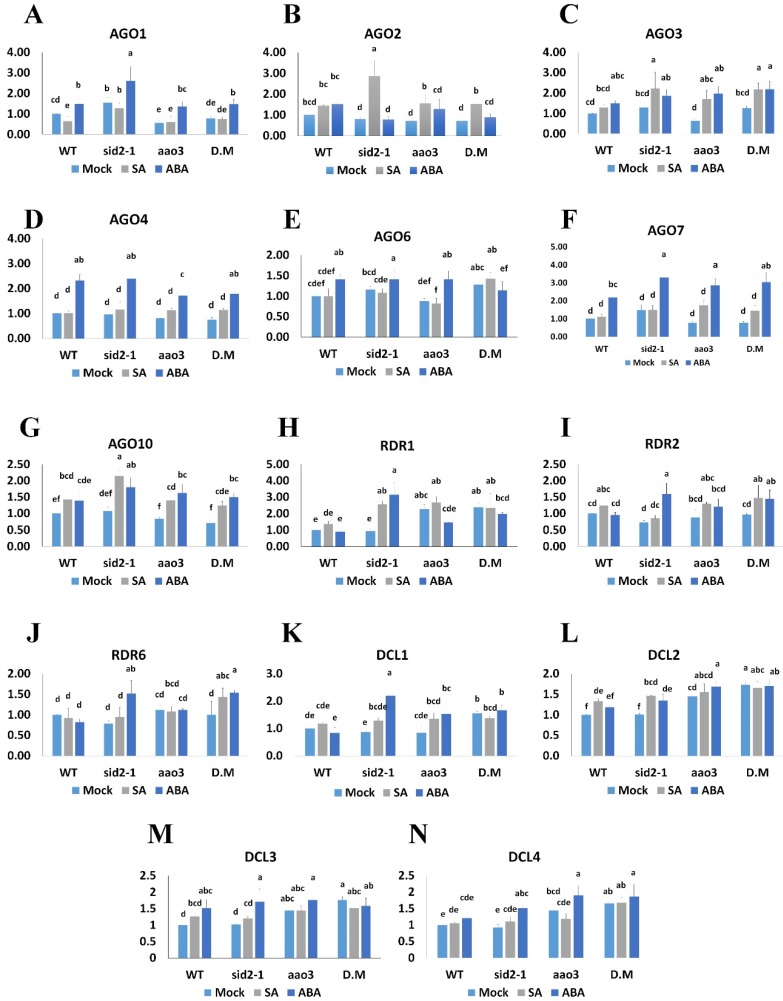
Effects of ABA and SA treatment on the relative expression (as determined by RT-qPCR) of (**A**) *AGO1*, (**B**) *AGO2*, (**C**) *AGO3*, (**D**) *AGO4*, (**E**) *AGO6*, (**F**) *AGO7*, (**G**) *AGO10*, (**H**) *RDR1*, (**I**) *RDR2*, (**J**) *RDR6*, (**K**) *DCL1*, (**L**) *DCL2*, (**M**) *DCL3*, and (**N**) *DCL4* in seedlings of the *Arabidopsis thaliana* wild type (WT), the SA mutant *sid2-1*, the ABA mutant *aao3*, and their double mutant (D.M) *sid2-1:aao3*. Six-day-old seedlings were treated with SA (50 µM), ABA (10 µM), or mock (0.1% EtOH); expression was determined by RT-qPCR 2 days later. Values are means + SD of three biological replicates, each carried out with three technical replicates. Analyses of variance (ANOVAs) were conducted to determine significant differences (*p* < 0.05); when ANOVAs were significant, the means for each gene were compared with a Duncan’s post hoc test; means that share a lowercase letter are not statistically different, while different letters denote for statistical differences at least 95% confidence.

Interestingly, SA exhibited negative effects on *AGO1* expression as SA treatment decreased *AGO1*, while SA mutants increased *AGO1* expression (Figure 2A). In contrast, ABA treatment enhanced *AGO1* expression in all lines, especially in *sid2-1* in which ABA content is higher than WT [7] (Figure 2A). In line with ABA effects, impairment of ABA in the single mutant reduced *AGO1* expression (Figure 2A).

Both SA treatment and ABA treatment caused marginal increases in the expression of *AGO2* in WT seedlings, but ABA treatment failed to increase *AGO2* expression in SA mutants (*sid2-1* or *sid2-1:aao3*) (Figure 2B). In contrast, SA treatment increased *AGO2* expression in both ABA and SA mutants, as well as in their double mutant. Because the impairment of SA synthesis diminished the effect of ABA, we inferred that ABA acts upstream of SA in inducing *AGO2* expression. In the mock-treated lines, *AGO2* expression was marginally lower in the single and double mutants than in the WT, which confirmed that both hormones enhance *AGO2* expression (Figure 2B). Similarly, *AGO3* and *AGO10* showed marginal increases following ABA or SA treatment of WT plants. Nevertheless, the effect of both hormones was more pronounced on single and double mutants than on the WT, leading to significant differences in the expression of both genes (Figure 2C,G). 

ABA treatment increased the expression of *AGO4*, *AGO6*, and *AGO7* in all lines except for expression of *AGO6* in the double mutant (Figure 2D–F). Expression of *AGO4*, *AGO6*, and *AGO7* was not altered by SA treatment or in the *sid2-1* mutant (Figure 2D–F). Therefore, we inferred that both hormones have similar positive effects on the expression of *AGO2*, -*3*, *-4*, and -*10*, while SA exerts a negative effect on the expression of *AGO1*.

In the RDR family, SA treatment of the WT seedlings marginally increased the expression of *RDR1* (Figure 2H) but did not affect the expression of *RDR2* or *RDR6* (Figure 2I,J). ABA treatment increased the expression of *RDR1*, *RDR2*, and *RDR6* only in *sid2-1* (Figure 2H–J).

The ability of ABA to induce *RDR1*, *RDR2*, and *RDR6* expression when SA synthesis was impaired indicated that the antagonism between ABA and SA influences the expression of these genes. 

In WT plants, SA treatment increased *DCL2* expression and marginally induced *DCL3* and *4* (Figure 2K–N), while ABA treatment of WT plants increased *DCL3* expression and marginally increased *DCL4* expression (Figure 2M,N). In the mock-treated *aao3* mutant, the expression of *DCL2*, -*3*, and -*4* was higher than in WT mock. However, SA or ABA treatment often increased the expression of these genes in single mutants, notably in *sid2-1* treated with ABA, which indicates that such genes are more sensitive to ABA than to SA. Hormone effects were absent in the double mutant, perhaps because the *DCL* expression levels were already substantially higher in the mock-treated double mutant than in the mock-treated WT. These results indicate that *DCL* genes might be regulated in parallel by several factors or hormones that exert clear effects under low ABA or SA conditions, suggesting that such factors/hormones might be antagonistic to both ABA and SA.

### 2.3. Susceptibility to BaMV Is Not Greater in the ABA/SA Double Mutant Than in the Single Mutants

**Figure 3:**
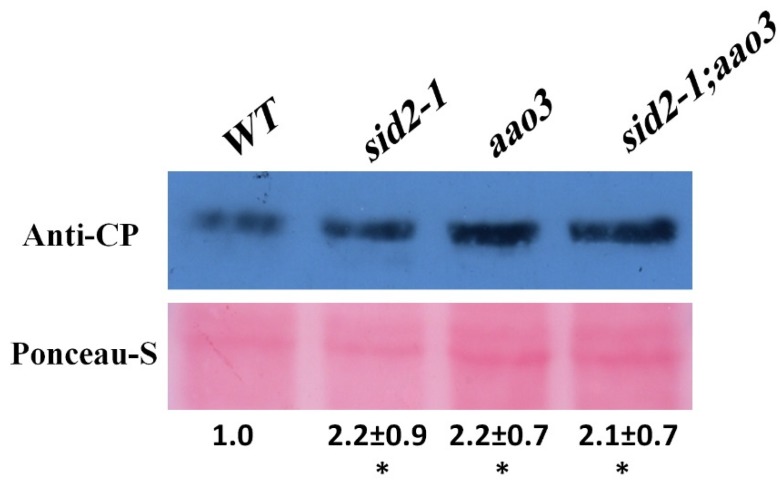
The susceptibility of wild type (WT) and ABA and SA mutant seedlings to BaMV. Six-day-old seedlings of the WT, *sid2-1*, *aao3*, and *sid2-1:aao3* were infected with a BaMV infectious clone (pKB) using Agrobacterium-mediated transformation (AGROBEST method). Total proteins were extracted 4 days later, and BaMV coat protein was quantified by Western blot analysis as indicated in the figure. The experiment was carried out in 3 biological replicates with similar results (Figure S2). Band densities (anti-CP and RuBisCO from Ponceau-S staining) were measured with Image J software, and the CP level in each line was normalized to the corresponding Rubisco band. Values are means ± SD of three biological replicates. An asterisk indicates a significant increase relative to the WT (which was set at 1.0) according to a one-sided Student’s *t*-test (*p* < 0.05).

Finally, we tested the effect of impaired SA and ABA synthesis on plant tolerance to BaMV infection. Using AGROBEST methodology (Agrobacterium-mediated transformation of BaMV infectious clone (pKB)) [3,79], WT, the SA mutant *sid2-1*, the ABA mutant *aao3*, and the SA-ABA double mutant *sid2-1:aao3* were transfected with pKB. As expected, BaMV CP levels were higher in the single mutants than in the WT (Figure 3). The BaMV CP level was not higher, however, in the double mutant than in the single mutants (Figure 3). These results indicate that the affected defense mechanism in the single mutants might be regulated in parallel by SA and ABA (Figure 3).

## 3. Discussion

The coexpression analyses conducted using PlantPan 2.0 provided a list of TFs that might be coexpressed with the RNA silencing-related genes of interest in this study. These TFs had been experimentally verified to be induced by SA and ABA treatments based on publicly available RNAseq data that PlantPan 2.0 and ExPath 2.0 collaboratively use [20,25]. The coexpression analyses showed that the selected TFs can be coexpressed with RNA silencing genes in response to various hormone treatments (Table 1). TFBSs in the promoter regions of the RNA silencing genes were then identified based on the experimentally verified *cis*-elements compiled in PlantPan 2.0.

Both ABA and SA have been reported to greatly affect the resistance to several viruses [10,11], including BaMV, PVX, and soybean mosaic virus in response to ABA [3,12]. With bamboo mosaic virus as an example, the results presented here suggest that certain ABA- or SA-related TFs regulate the expression of the RNA silencing genes, which may explain how both hormones influence resistance to viruses through the RNA silencing pathway. 

In response to ABA, several TFs are coexpressed and act hierarchically; their dynamic binding to the promoters of target genes defines plant responses to the stress [80]. Song et al. (2016) found that highly upregulated genes are often targeted by several TFs through top-up building, while downregulated genes are associated either with static binding by TFs or with downregulated TF binding. It follows that the hierarchical activity of the coexpressed ABA-related TFs identified in Table 1 can promote coordinated regulation of the ABA-regulated RNA silencing genes. Such dynamic binding activity can also be expected for the SA-related TFs on their target genes. 

The RNA silencing pathways are partially controlled by several hormones/networks in order to fine-tune plant responses to various biotic/abiotic stresses. Hormone pathways such as those for SA, ABA auxin, and ethylene were previously reported to partially regulate a few components in the RNA silencing pathways [3,13,14,16,81,82]. Although antagonism is the only previously reported relationship that directly governs the interactive effects of the SA and ABA pathways, the current study shows that their regulatory effects on the expression of the RNA silencing genes are similar for some genes, antagonistic for others, and hierarchical for *AGO2* (Figure 2). The expression of *AGO1* is probably a subject of such antagonism because the SA biosynthesis gene *ICS1* is highly upregulated in the *aao3* mutant [7], which might explain the downregulation of *AGO1* in *aao3*. Antagonism between ABA and SA was also observed for their regulation of the *RDR* genes in that ABA enhanced their expression only in SA mutants (Figure 2H–J). 

Both hormones had marginal but positive effects on the expression of *AGO2*, *AGO3*, *AGO10*, *DCL2*, *DCL3*, and *DCL4* in WT seedlings (Figure 2). However, such effects become more pronounced when a mutant of either pathway was treated with the hormone of the other pathway; this was the case for most of these genes when *sid2-1* was treated with ABA, and for *AGO2*, *AGO3*, and *AGO10* when *aao3* was treated with SA. The inability of ABA to induce *AGO2* in the SA mutant indicates that ABA acts upstream of SA in inducing *AGO2* and represents a rare example of SA-dependency for an ABA-regulated gene (Figure 2B). It was previously reported that SA treatment only slightly increased *AGO2* expression in WT plants, but substantially increased *AGO2* expression in 2b-transgenic lines (cucumber mosaic virus 2b protein) [83]. The existence of TFs that are positively regulated by SA and that bind to some *AGO* promoters suggests that SA may enhance the expression of *AGO4*, *AGO6*, and *AGO7*, but the expression of such genes is probably spatiotemporal regulated. In previous studies, SA increased *AGO1* expression during leaf senescence [70,84], but SA reduced *AGO1* expression in seedlings in the current study (Figure 2). Thus, the effect of SA on *AGO1* can be positive or negative depending on plant age or tissue (Figure 1). The regulatory effect of ABA on some genes may also be age-dependent [85]. ABA treatment did not increase *AGO7* expression in 35-day-old *A. thaliana* plants in our previous study [3], but did so in the 8-day-old seedlings of the current study (Figure 2F). This suggests that the effect of ABA could be spatiotemporal and/or indirect. 

None of the mutants tested, including the double mutant, showed a substantial decrease in the expression of any of the genes in the seedling stage (eight days old) (Figure 3). This observation supports the notion that the RNA silencing pathway might be controlled by several networks involved in several cellular processes, such that plants cannot afford a complete shutdown of this pathway. As a consequence, the expression levels of many of those genes are evidently maintained at some minimum level by multiple factors. 

The expression of *AGO1*, *2*, *3*, and *10* was marginally downregulated in the *aao3* and *sid2-1* mutants (Figure 2A,B,C,G). Except for *AGO10*, mutants of all of these *AGO*s affect the susceptibility of *A. thaliana* to BaMV [3]. None of these *AGO*s showed enhanced downregulation in the double mutant, which suggests that there are other complementary factors/hormones that help maintain a minimum level of *AGO* gene expression in the absence of ABA or SA. These other factors could include the antagonistic hormones jasmonic acid, ethylene, or auxins [86], which may regulate the expression of some RNA silencing genes through their related TFs (Table S1). In addition, the enhanced susceptibility to BaMV in the single or double mutants at the seedling stage suggests that other defense mechanisms are also influenced by the impairment of ABA or SA pathways. Callose deposition, which limits virus spread, is decreased when ABA or SA levels are low [11]. ROS accumulation, which also contributes to basal immunity against several viruses, is increased in the *sid2-1* mutant, but ROS levels were lower in the ABA signaling mutant *abi4-1* than in the WT [87,88]. The overall outcome of defense against BaMV in *A. thaliana* seedlings appears to be equal among single and double mutants.

In conclusion, ABA and SA enhance plant defense to RNA viruses largely via the antiviral RNA silencing pathway, i.e., both hormones increase the expression of those genes in the RNA silencing pathway that are required for Arabidopsis resistance to BaMV. The coexpression analyses identified several ABA and SA TFs that may regulate the expression of those genes. Other candidate TFs regulated by other hormones such as ethylene or jasmonic acid also suggest that the RNA silencing pathway is a hub tuned by hormones in response internal or external stimuli. The effect of hormones on RNA silencing may not be equal or similar; for example, the expression of several *AGO*s was influenced more by ABA than by SA. The effects of ABA and SA on the RNA silencing genes depend on the individual gene: in some cases, the effects are similar, and in other cases they are redundant, hierarchical, or antagonistic. It also seems that the effect of hormones on the RNA silencing pathway is influenced by plant age and tissue. Additional research is therefore needed to understand how hormones regulate the RNA silencing pathway during different stages of plant growth and in response to different stresses.

## 4. Materials and Methods 

### 4.1. Coexpression and Promoter Analyses

PlantPan 2.0 was used to identify TFs that are coexpressed with genes in the AGO, DCL, and RDR families [20]. By selecting hormone treatments and/or environmental stress conditions, we obtained lists of TFs that are coexpressed with the queried gene as indicated by a Pearson correlation coefficient with a *p*-value > 0.8 (Table S1). To obtain a shortlist of TFs that are regulated only by SA or ABA, GO and functional descriptions of these TFs were queried manually in the Plant Transcription Factor Database v4.0 http://planttfdb.cbi.pku.edu.cn/ [89]. All TFs regulated by ABA or SA along with the promoters that the TFs probably bind to are listed in Table 1. 

Regions 2000 bp upstream of the transcription start sites (TSSs) containing the promoters of *AGO*, *DCL*, and *RDR* family genes were subjected to promoter analyses using the Plant Promoter Analysis Navigator in PlantPan 2.0 [20]. The TFs and their binding sites within the promoter regions were visualized using PlantPan 2.0. 

### 4.2. Plant Materials

The following *A. thaliana*lines were used; wild type (Col-0), SA-biosynthesis mutant (*sid2-1*), ABA-biosynthesis mutant (*aao3*), and their double mutant (*sid2-1:aao3*). Seeds of these lines were germinated and grown in liquid MS medium at 20 °C under a 16/8 h photoperiod in 6-well plates until they were 6 days old, at which time hormone treatments were applied.

### 4.3. Hormone Treatments

When seedlings were 6 days old, the liquid MS medium was replaced with fresh MS containing ABA (10 µM), SA (50 µM), or mock (0.5% EtOH in ddH_2_O). Two days after treatment, 10 seedlings from each line (WT, *sid2-1*, *aao3*, and *sid2-1:aao3*) were collected for RNA extraction and cDNA synthesis. 

### 5.4. RNA Extraction and Real-Time Quantitative PCR (RT-qPCR) 

Total RNA was extracted using TRIzol following the manufacturer’s instructions. First-strand cDNA was generated with the Superscript III kit (Invitrogen), and RT-qPCR was carried out with SYBR green (Roche) using ΔΔ*C*_t_ methodology. *Actin* was used as a reference gene as described previously [3,90]. The RT-qPCR primers used in this study are listed in Table S3. One-sided Student’s *t*-tests (*p* < 0.05) were used to determine whether the expression level of each gene in each line was upregulated or downregulated relative to the mock-treated WT.

### 4.5. Protein Analyses

Six-day-old seedlings of the WT, *sid2-1*, *aao3*, and *sid2-1:aao3* were inoculated with the pKB clone of BaMV [91] using the AGROBEST method as described previously [3,79]. Total proteins were extracted 4 days after inoculation as described previously [3]. BaMV coat protein (CP) was detected as described previously [23,90]. The experiment was carried out in 3 biological replicates with similar results (Figure S2).

### 5.6. Statistical Analysis

Treatments were applied to seedlings arranged in completely randomized blocks. RTqPCR data (means ± SD) were subjected to two-way analyses of variance (ANOVAs) to compare the effects of hormone treatments and plant lines on the expression of each gene. When an ANOVA was significant (*p* < 0.05), means were compared with a Duncan’s post hoc test. Different letters denote for statistical differences at least 95% confidence. The CoStat package, version 6.451, was used for statistical analysis.

## Figures and Tables

**Table 1:** Transcription factors that are involved in the regulation of ABA or SA responses and that can be coexpressed with silencing-related genes: TF: Transcription factor; Acc. No.: Accession number; ABA: abscisic acid; JA: Jasmonic acid; SA: salicylic acid; Et: Ethylene. References: [29] (Nakashima et al., 2006), [30] (Delmas et al., 2013), [31] (Acevedo-Hernandez et al., 2005), [32] (Kaliff et al., 2007), [33] (Shkolnik-Inbar and Bar-Zvi, 2010), [34] (Shu et al., 2013), [35] (Feng et al., 2014), [36] (Lopez-Molina et al., 2001), [37] (Carles et al., 2002), [38] (Bensmihen et al., 2002), [39] (Lopez-Molina et al., 2002), [40] (Finkelstein et al., 2005), [41] (Kim et al., 2012), [42] (Seo et al., 2010), [26] (Jensen et al., 2008), [27] (Wu et al., 2009), [43] (Lechner et al., 2011), [44] (Johannesson et al., 2003), [45] (Jiang and Yu, 2009), [46] (Sakuma et al., 2002), [47] (Lee et al., 2010), [48] (Kim et al., 2011), [49] (Tang et al., 2013), [50] (van Hengel et al., 2004), [51] (Soderman et al., 1996), [52] (Valdes et al., 2012), [53] (Chinnusamy et al., 2003), [54] (Yamamoto et al., 2009), [55] (Brocard-Gifford et al., 2003), [56] (Reyes and Chua, 2007), [57] (Tran et al., 2004), [58] (Yanhui et al., 2006), [59] (Li et al., 2006), [60] (Kang and Singh, 2000), [61] (Kang et al., 2003), [62] (Johnson et al., 2003), [63] (Zander et al., 2010), [64] (Fonseca et al., 2010), [65] (Thibaud-Nissen et al., 2006), [66] (Choi et al., 2010), [67] (Zhou et al., 2000), [68] (Kang and Klessig, 2005), [69] (Duan et al., 2007), [70] (Robatzek and Somssich, 2001), [71] (Yu et al., 2001), [72] (Lai et al., 2008), [73] (Chen et al., 2010), [74] (Hu et al., 2013), [75] (Chen et al., 2013), [76] (Zhang et al., 2007), [77] (Scarpeci et al., 2013), [78] (Li et al., 2013).

TF	Acc. No.	Promoters of																TF Family	Regulation	GO/Functional Description	Reference
		AGO									RDR			DCL							
		1	2	3	4	5	6	7	9	10	1	2	6	1	2	3	4				
ABI3	AT3G24650	●							●	●				●				B3	ABA	ABA-activated signalling pathway - response to ABA	[29,30]
ABI4	AT2G40220														●			ERF	ABA	ABA-activated signalling pathway	[31,32,33,34,35]
ABI5	AT2G36270	●								●								bZIP	ABA	ABA-activated signalling pathway - response to ABA	[29,36,37,38,39,40]
ANAC062	AT3G49530			●														NAC	ABA	Plays a regulatory role in ABA-mediated drought-resistance. Mediates induction of pathogenesis-related (PR) genes independently of salicylic signalling in response to cold	[41,42]
ATAF1	AT1G01720	●													●	●		NAC	ABA	Negative regulation of ABA-activated signalling pathway	[26,27]
AtHB33	AT1G75240	●	●					●				●			●		●	ZF-HD	ABA	Repressed by ABA and ARF2, Regulators in the ABA signal pathway that confers sensitivity to ABA in an ARF2-dependent manner.	[27]
ATHB5	AT5G65310												●					HD-ZIP	ABA	Probable transcription factor that acts as a positive regulator of ABA-responsiveness, mediating the inhibitory effect of ABA on growth during seedling establishment. Binds to the DNA sequence 5′-CAATNATTG-3′.	[27]
AT-HSFA9	AT5G54070					●												HSF	ABA	A member of Heat Stress Transcription Factor (Hsf) family. Not responding to heat stress. Is regulated by the seed-specific transcription factor ABI3. In turn, it regulates other heat stress proteins including Hsp17.4-CI, Hsp17.7-CII and Hsp101 during seed maturation.	[44]
DREB19	AT2G38340		●									●		●		●		ERF	ABA	Induced by ABA treatment. Transcriptional activator that binds specifically to the DNA sequence 5′-[AG]CCGAC-3′. Binding to the C-repeat/DRE element mediates ABA-inducible transcription	[46]
DREB2	AT5G05410		●		●				●			●		●	●	●		ERF	ABA	the ABA-dependent pathway plays a positive role in the osmotic stress-responsive expression of DREB2A	[47,48]
FHY3	AT3G22170				●													FAR1	ABA	FHY3 and FAR1 are positive regulators of ABA signalling and provide insight into the integration of light and ABA signalling	[49]
GL2	AT1G79840												●					HD-ZIP	ABA	The expression patterns of arabinogalactan-protein AtAGP30 and GLABRA2 reveal a role for ABA in the early stages of root epidermal patterning.	[50]
HB-7	AT5G46880					●							●					HD-ZIP	ABA	NDUCTION: By water deficit, by ABA and by salt stress	[51,52]
ICE1	AT3G26744		●		●			●	●	●		●		●	●	●	●	bHLH	ABA	INDUCTION: By high-salt stress, cold stress and ABA treatment.	[53]
LEC1	AT1G21970					●							●					NF-YB	ABA	ABA-activated signalling pathway	[55,57]
MYB33	AT5G06100				●			●						●				MYB	ABA	positive regulation of ABA-activated signalling pathway	[56]
NAC3	AT3G15500			●														NAC	ABA	Strongly induced by high salinity. Slightly up-regulated by drought, ABA and jasmonic acid. Not induced by cold treatment.	[57]
WRKY2	AT5G56270												●					WRKY	ABA	Transcription factor. Regulates WOX8 and WOX9 expression and basal cell division patterns during early embryogenesis. Interacts specifically with the W box (5′-(T)TGAC[CT]-3′), a frequently occurring elicitor-responsive cis-acting element. Required to repolarize the zygote from a transient symmetric state	[45]
ATHB16	AT4G40060										●							HD-ZIP	ABA	MATH/BTB CRL3 receptors target the homeodomain-leucine zipper ATHB6 to modulate ABA signalling.	[43]
MYB59	AT5G59780										●							MYB	SA	Isoform MYB59-1 is induced by JA, SA, gibberellic acid, and ethylene	[58,59]
MYB65	AT3G11440							●										MYB	SA	response to SA	[58]
OBP1	AT3G50410	●	●		●			●	●	●		●		●	●	●		bZIP	SA	Induced by SA, Constitutively expressed in the whole plant	[59]
TGA3	AT1G22070					●												bZIP	SA	systemic acquired resistance, SA mediated signalling pathway	[62,65,66,67,68]
TGA4	AT5G10030	●			●	●		●	●	●		●		●	●	●	●	bZIP	SA	Binding to the as-1-like cis elements mediate auxin- and SA-inducible transcription. May be involved in the induction of the systemic acquired resistance (SAR) via its interaction with NPR1.	[60,67]
WRKY1	AT2G04880	●	●						●			●					●	WRKY	SA	SA-mediated signalling pathway	[69]
WRKY15	AT2G23320																●	WRKY	SA	Induced by SA	[71]
WRKY21	AT2G30590									●				●			●	WRKY	SA	Induced by SA	[71]
WRKY26	AT5G07100										●							WRKY	SA	Induced by SA	[71,76]
WRKY3	AT2G03340	●	●			●			●	●		●		●	●	●	●	WRKY	SA	induced by SA and during leaf senescence	[70,71]
WRKY30	AT5G24110			●														WRKY	SA	response to SA	[77]
WRKY4	AT1G13960								●	●				●	●	●	●	WRKY	SA	INDUCTION: By biotic and abiotic stresses such as pathogen infection, SA, JA, ACC	[72]
WRKY54	AT2G40750										●							WRKY	SA	WRKY70 and WRKY54 co-operate as negative regulators of stomatal closure and, consequently, osmotic stress tolerance in Arabidopsis, suggesting that they have an important role, not only in plant defence, but also in abiotic stress signalling. WRKY70 and WRKY54 are positive regulators of plant defence, and co-operate as negative regulators of SA biosynthesis and senescence.	[78]
OBP3	AT3G55370								●									bZIP	SA- JA	Induced by SA, Repressed by JA	[60,61]
TGA2	AT5G06950	●	●			●		●	●	●		●		●	●	●	●	bZIP	SA- JA/Et	Required to induce the systemic acquired resistance (SAR) via the regulation of pathogenesis-related genes expression	[62,63,64,65]
TGA5	AT5G06960		●		●			●	●	●		●		●		●	●	bZIP	SA- JA/Et	May be involved in the induction of the systemic acquired resistance (SAR) via its interaction with NPR1.	[63,67,68]
TGA6	AT3G12250	●	●			●		●	●	●		●		●	●	●	●	bZIP	SA- JA/Et	May be involved in the induction of the systemic acquired resistance (SAR) via its interaction with NPR1.	[63,67,68]
WRKY8	AT5G46350			●														WRKY	SA/ABA	Induced by wounding, ABA, SA, H2O2 and infection with P.syringae pv. tomato DC3000 and B.cinerea	[73,74,76]

## Supplementary Materials

Supplementary materials can be found at https://www.mdpi.com/1422-0067/20/10/2538/s1. Figure S1. Transcription factor binding sites of TFs on promoters with abundant *cis*-regulatory elements: The TFs HB33, ICE1, OBP1 (TGA1), TGA2, TGA3, TGA4, TGA5, and TGA6 have several binding sites for the promoters of RNA silencing genes. Transcription factors TGA1 (OBP1), 2, 3, 4, 5, and 6 bind to the same TF-motif TGACG (TF_motif_seq_0271) [92,93,94,95]. PlantPan 2.0 was used for promoter analysis and TFBS visualization. Figure S2. The susceptibility of wild type (WT) and ABA and SA mutant seedlings to BaMV infection. Table S1. Lists of TFs that are coexpressed with the RNA silencing genes (AGO, DCL, and RDR gene families) in response to the indicated hormone treatments and environmental stresses. TFs were included if their Pearson correlation coefficients were *p*-values > 0.9 (except that TFs for *AGO5* and *RDR1* were included if their correlation coefficients were >0.6). Table S2. Characteristics of the public microarray data compiled in ExPath 2.0 and used by PlantPan 2.0 for coexpression analyses. Table S3. Primers used in the study.
