# Supplementary figures and images for: Effects of Abscisic Acid and Salicylic Acid on Gene Expression in the Antiviral RNA Silencing Pathway in Arabidopsis
Source: Int J Mol Sci. 2019 May 23;20(10):2538. doi: 10.3390/ijms20102538 (PMC6566719; doi:10.3390/ijms20102538)

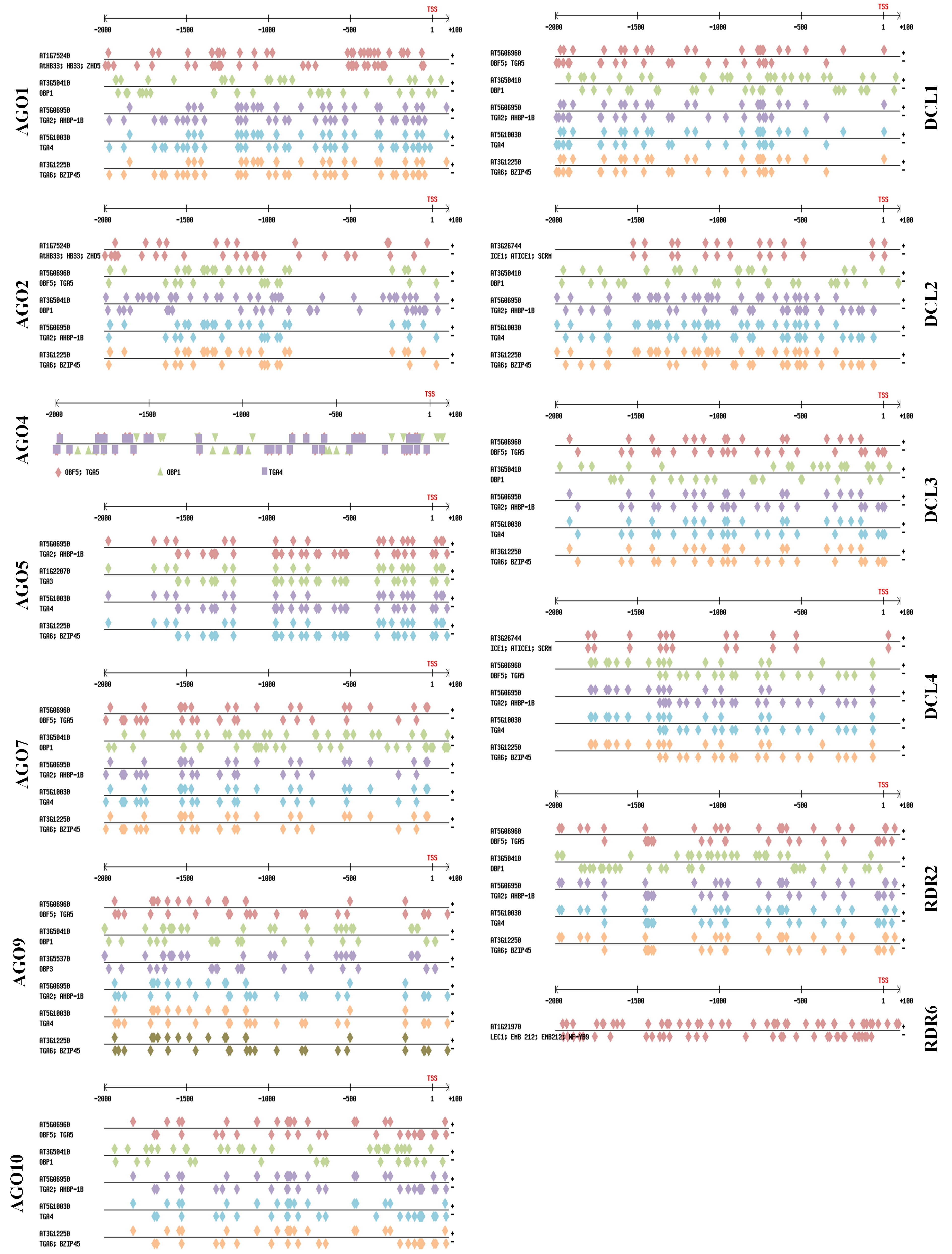

Supplement: Supplementary file 1 [file ijms-20-02538-s001.zip › S1.tif]

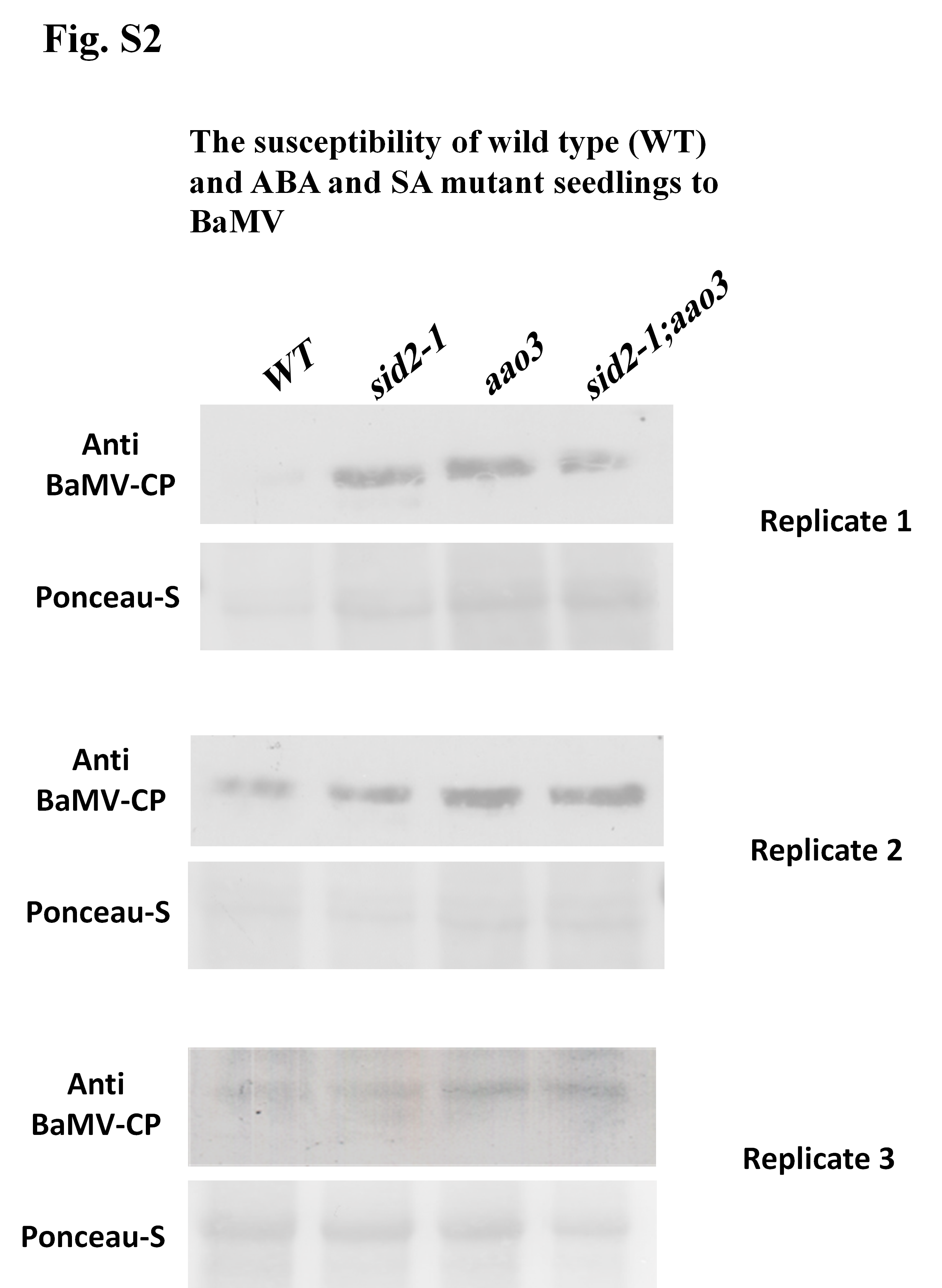

Supplement: Supplementary file 1 [file ijms-20-02538-s001.zip › S2.tif]
